# Longitudinal and concurrent links between memory span, anxiety symptoms, and subsequent executive functioning in young children

**DOI:** 10.3389/fpsyg.2014.00443

**Published:** 2014-05-16

**Authors:** Laura Visu-Petra, Oana Stanciu, Oana Benga, Mircea Miclea, Lavinia Cheie

**Affiliations:** ^1^Developmental Psychology Lab, Department of Psychology, Babeş-Bolyai UniversityCluj-Napoca, Romania; ^2^Department of Applied Mathematics and Computer Science, University of GhentGhent, Belgium; ^3^Department of Psychology, Applied Cognitive Psychology Center, Babeş-Bolyai UniversityCluj-Napoca, Romania

**Keywords:** executive functions, working memory, short term memory span, anxiety, inhibition, shifting, young children

## Abstract

It has been conjectured that basic individual differences in attentional control influence higher-level executive functioning and subsequent academic performance in children. The current study sets out to complement the limited body of research on early precursors of executive functions (EFs). It provides both a cross-sectional, as well as a longitudinal exploration of the relationship between EF and more basic attentional control mechanisms, assessed via children's performance on memory storage tasks, and influenced by individual differences in anxiety. Multiple measures of verbal and visuospatial short-term memory (STM) were administered to children between 3 and 6 years old, alongside a non-verbal measure of intelligence, and a parental report of anxiety symptoms. After 9 months, children were re-tested on the same STM measures, at which time we also administered multiple measures of executive functioning: verbal and visuospatial working memory (WM), inhibition, and shifting. A cross-sectional view of STM development indicated that between 3 and 6 years the trajectory of visuospatial STM and EF underwent a gradual linear improvement. However, between 5 and 6 years progress in verbal STM performance stagnated. Hierarchical regression models revealed that trait anxiety was negatively associated with WM and shifting, while non-verbal intelligence was positively related to WM span. When age, gender, non-verbal intelligence, and anxiety were controlled for, STM (measured at the first assessment) was a very good predictor of overall executive performance. The models were most successful in predicting WM, followed by shifting, yet poorly predicted inhibition measures. Further longitudinal research is needed to directly address the contribution of attentional control mechanisms to emerging executive functioning and to the development of problematic behavior during early development.

## Introduction

During the past decades, the importance of investigating the early development of executive functions (EFs) has been reinforced by a growing body of evidence linking preschool EFs measures to emerging academic success (see Willoughby et al., 2012a, for a recent review), to social competence during early school years (Ciairano et al., 2007; Razza and Blair, 2009), and also to internalizing and externalizing symptoms (Thorell and Wåhlstedt, 2006; Brocki et al., 2010; Hughes and Ensor, 2011). This endeavor was previously constrained by the limited methodological repertoire allowing researchers to track EF progress across successive developmental periods. Recently, the gap has been addressed by developing a wide range of child-friendly tasks for measuring EF during early development (see Carlson, 2005; Garon et al., 2008 for reviews), with evidence of relatively reliable psychometric properties for this age span (Miller et al., 2012; Willoughby et al., 2012a).

However, the early developmental course and changing structure of executive functioning is not yet fully captured by the limited body of prospective longitudinal data (but see Hughes et al., 2010; Röthlisberger et al., 2012; and Willoughby et al., 2012b, for notable exceptions), most of the research in the field being still cross-sectional. Also, the fundamental prerequisites from the first years of life have not yet been convincingly linked to the intricate nature of later EF, which has been regarded as the most complex form of high-level human cognition (Salthouse, 2005). Moreover, executive control is also determined by, and influential for, emotion-cognition interactions (Pessoa, 2008), which generate stable predispositions in information processing mechanisms (e.g., Pine, 2007), regarded as early cognitive vulnerability markers for a variety of psychopathologies such as internalizing disorders (Ingram and Price, 2010). Further longitudinal studies complementing the limited existing literature (e.g., Riggs et al., 2004; Hughes and Ensor, 2011; Tillman et al., 2013) are necessary in order to construct true developmental models of how early EF and socio-emotional processes interact to generate problematic behavior and cognitive vulnerabilities to psychopathology.

### Early EF development and its precursors

With regards to the early developmental trajectory of executive control, initial models argued for the predominant role of one EF, such as *inhibitory control* (Diamond and Gilbert, 1989; Dempster, 1992; Barkley, 1997; Carlson et al., 1998) or *working memory* (WM; Pascual-Leone, 1970; Case, 1985; Morton and Munakata, 2002). A step forward consisted in considering both inhibition and WM as central to EF development (Diamond, 1991; Roberts and Pennington, 1996). The seminal model proposed by Miyake and collaborators (2000) identified three “independent, yet interdependent” EF dimensions: updating of WM representations, inhibition, and shifting. This model was later refined and the identity of inhibition as a distinct factor was questioned. Inhibition subsequently came to be related to common variance in EF tasks (e.g., Friedman et al., 2008; Miyake and Friedman, 2012). The third dimension, *shifting* was defined as the ability to flexibly shift among distinct but related aspects of a given stimulus or task set (Zelazo and Müller, 2002). The tripartite model of EF has been partially confirmed by latent variables analyses conducted in older children samples (Lehto et al., 2003; Huizinga et al., 2006; but see Lee et al., 2012; Van der Ven et al., 2012, for failures to replicate this structure). However, similar studies with preschool children have pointed toward a more unitary structure of EF (Wiebe et al., 2008, 2011; Hughes et al., 2010; Willoughby et al., 2010), although a two-dimensional structure, integrating WM and inhibition as separate yet related factors, was also found (Lerner, 2012; Miller et al., 2012).

Our study was designed to investigate the early developmental interrelations between individual differences in attentional control, memory storage, anxiety symptoms, and subsequent executive functioning (WM, inhibition, shifting) during preschool years. Therefore, we will now review the available evidence on the precursors and subcomponents of these three EF dimensions. The few existing longitudinal studies have generally overlooked how preschool EFs are linked to more basic precursors, such as attentional or memory processes. However, in the literature, there have been some theoretical conjectures regarding these elementary forms of EF. One of the most well-articulated frameworks has been proposed by Garon and collaborators (2008). The authors argue that EF components are built upon simpler cognitive skills and represent the coordination of these basic skills, essentially occurring after the age of three. As a potential candidate, they suggested that the “maturation of attentional capacity forms a foundation for the development of EF abilities during the preschool period, and, in fact, may be the source of common variance underlying various EF skills” (p. 35). Simple span tasks have been proven to rely on individuals' ability to consistently focus and control their attention in order to maintain or suppress information (Engle, 2002) and therefore, might represent an ideal context in which to assess early attentional precursors of EF.

WM processes relate to the updating and active use of temporarily available information. Complementary to this definition, short-term memory (STM) represents the temporarily increased availability of information in memory that may be used to carry out various types of mental tasks (Cowan et al., 1999). The model proposed by Baddeley and Hitch (1974; see also Baddeley, 2000) represents the preferred theoretical framework in which WM development is studied. Various *simple memory span* tasks have been used to measure the two STM storage systems: the phonological loop, and the visuospatial sketchpad. These “slave” systems feed their input into the central executive, a system involved in supervising and adjusting the control of memory contents. Almost all STM measures present a steady *increase* from the preschool years until adolescence (Gathercole et al., 2004; Alloway et al., 2006). *Complex memory span* tasks involve both maintenance and manipulation of information, and are considered measures of WM capacity. The memory components corresponding to the central executive, the phonological loop, and the visuospatial sketchpad appear to resemble the adult tripartite distinction, and to be evident in children as young as four (Alloway et al., 2006). However, it is important to mention that there are strong competing models (Engle et al., 1999; Cowan, 2001; Barrouillet et al., 2004), most of them focusing on the importance of attentional control mechanisms involved in both information storage and processing. Further research on longitudinal interrelations between early aspects of attentional control, memory storage and processing could benefit the integration of the multiple theoretical accounts of WM development.

Two different perspectives could be proposed regarding the involvement of STM processes in WM development and in relation to EF tasks, in general. One of them considers that the active manipulation of information is essential to WM/EF processes (Miyake et al., 2000). Hence, tasks requiring only memory span and which lack this dimension would share only non-executive variance with WM/EF tasks (Lerner, 2012). Another perspective suggests that both simple span and WM tasks share common attentional control demands, and thus their covariance would rely on both executive and non-executive processes. More specifically, WM processes reflect the functioning of the central attention system and its role in the coordination of the systems involved in storage (Garon et al., 2008). The authors argue for the need to conduct longitudinal studies using both complex and simple span tasks in order to “draw conclusions about whether complex WM tasks build upon simpler memory abilities and skills” (p. 40). Beyond its importance for WM development, STM performance could also be predictive for performance in other EF tasks, such as *inhibitory control*. When analyzing the early development of inhibitory control, the focus is mainly on executive inhibitory processes, defined as processes for intentional control or response suppression in the service of higher order or longer-term goals (Nigg, 2000). Friedman and Miyake (2004) empirically differentiated simple *response suppression*, which refers to simply withholding a pre-potent response, from *attentional control/response conflict*, which encloses the inhibition of an internally represented rule/response set interfering with the ability to engage and implement a new rule/response. This distinction was confirmed in a study with preschoolers (Espy and Bull, 2005) showing that their performance on response conflict, but not on response suppression measures, was related to their simple spans, probably due to a common reliance on attention control mechanisms. Therefore, the current study set out to investigate the contribution of simple span (verbal or visuospatial) to WM, inhibition (response suppression and verbal and motor response conflict), and shifting in a preschool sample. However, it is important to note that Lerner (2012) failed to find evidence for the proposed dissociation between response suppression and attention control/response conflict in children. A similar less clear-cut distinction between the two inhibitory control dimensions was recently evidenced in a cross-cultural study with preschoolers (Cheie et al., 2014). As an alternative account, Diamond and Kirkham (2005) hypothesized that a common mechanism, called *attentional inertia* (a focus on the same, previously-relevant aspect of one stimulus, even when contextual demands are changing), would be responsible for children's inappropriate responses across various inhibitory and shifting tasks.

Although many tasks have been developed for measuring *shifting* in older children (e.g., Anderson et al., 2000; Jacques and Zelazo, 2001), it is much more difficult to identify comparable tasks for use in the preschool population (Lerner, 2012). In this population, the Dimensional Change Card Sort task (DCCS; Zelazo et al., 2003) has been extensively used to evaluate attention shifting. During task unfolding, children are presented with two target cards (e.g., a blue rabbit and a red boat) and subsequently requested to sort a series of bivalent test cards according to one dimension (e.g., color; the pre-switch phase). After becoming habituated with this dimension, children are asked to sort the same types of test cards according to another dimension (e.g., shape; the post-switch phase). Perseveration on an initial response set shows both the low memory strength of the new mental set (Munakata, 2001), and the reduced ability to inhibit interference from the initial mental set (Diamond et al., 2005). A shifting task either simply involves the coordination of these subordinated skills (Chevalier et al., 2012), or it represents a distinct process acting upon these skills and creating a modification in the original representation of the stimuli (Garon et al., 2008). A modified version of the DCCS was created, using emotional stimuli (facial expressions); the two sorting criteria were emotional expression (happy vs. sad) and gender (Qu and Zelazo, 2007). Children performed significantly better on the emotional faces version (with facilitative effects only in the case of happy faces), suggesting that positive stimuli might promote cognitive flexibility. Since one of our research questions was related to the impact of individual differences in anxiety on EF performance, we constructed an emotional DCCS version (Em-DCCS) similar to this emotional faces version. For this version, we used schematic depictions of facial emotional expressions (sad or happy faces) similar to the ones used by Hadwin and collaborators (2003) in their investigation of anxiety-related biases in visual search. The choice of schematic faces over real emotional expressions was also done in order to eliminate potential cultural effects related to the recognition of facial affect (Posner et al., 1994). The task requires children to switch in the post-shift phase from a neutral judgment (color) to a judgment of emotion (happy or sad faces). Our investigation extends the individual differences direction proposed by Qu and Zelazo (2007) by attempting to replicate the facilitative effect of positive faces on shifting performance, and by relating it to individual differences in trait anxiety. This has been associated with biases in the processing of stimuli with positive versus negative emotional valence in both adults (Chen et al., 2012) and young children (Visu-Petra et al., 2010).

### The role of individual differences in anxiety

From an early age, individual differences in anxiety have been shown not only to influence information processing patterns in contexts in which stimuli with emotional valence are present (Pine, 2007; Hadwin and Field, 2010), but also in contexts which lack such emotional information, especially tasks with higher levels of executive demands (see Visu-Petra et al., 2013a, for a review). The explanation of the relationship between individual differences in anxiety and impaired EF has been via the detrimental effects of anxiety on attentional control. This is reflected in the most influential explanatory framework regarding the anxiety—cognitive functioning relationship offered by the Attentional Control Theory (ACT; Eysenck et al., 2007). The theory predicts that in high-anxious individuals, anxiety-related worrisome thoughts interfere with their task-goals, requiring the activation of auxiliary processes and strategies. Accordingly, this concurrent resource activation is mostly evident in decreased performance *efficiency*, as more time and effort are required to complete a task, or to attain a given performance level. Yet, it can also be observed in terms of performance *effectiveness* (response accuracy), especially when the task is more challenging. A compelling body of evidence supports these predictions (see Eysenck and Derakshan, 2011, for a review), confirming that the anxiety-related depletion of resources impedes attention control, diminishing high-anxious individuals' EF (i.e., inhibition, shifting, and updating) performance.

Regarding the impact of anxiety upon preschoolers' STM, predictions are ambivalent. Preliminary evidence shows that, in line with related findings in older children (Hadwin et al., 2005), young children's simple span efficiency and, under certain circumstances, their accuracy, are affected by high trait anxiety levels (Visu-Petra et al., 2009, 2011). Trait anxiety was a longitudinal negative predictor of 3–6 year-old children's verbal STM performance accuracy, as well as efficiency of response, as indicated by a microanalysis of their response time segments (Visu-Petra et al., 2009). Another study revealed that while performance in the visuospatial span tasks did not differ between high-anxious and low-anxious preschoolers, high-anxious 3–7 year-olds displayed an inferior performance on the verbal simple and complex span measures (Visu-Petra et al., 2011). The findings also indicated that on simple span tasks, high-anxious preschoolers displayed efficiency impairments only, while both efficiency and accuracy of response were affected in the verbal WM tasks.

Although the developmental literature directly investigating the effects of anxiety upon EF is scarce (see Visu-Petra et al., 2013b for a review), the existent findings partially support the ACT predictions regarding anxiety's detrimental influence. Specifically, child anxiety has been found to disrupt inhibition efficiency (see Mueller, 2011, for a review), with a cross-cultural study in preschoolers identifying a greater impact of anxiety on performance efficiency in tasks requiring response conflict, compared to simple response suppression (Cheie et al., 2014). In a context requesting switching between neutral and emotional judgments, higher levels of trait anxiety were found to impair children's performance (Mocan et al., 2014). Several studies have also identified the negative impact of anxiety upon memory updating in younger and older children (e.g., Hadwin et al., 2005; Ng and Lee, 2010; Visu-Petra et al., 2011; Owens et al., 2012). Interestingly, the bidirectional nature of the link between anxiety and EF was recently documented via a longitudinal study that relates EF progress during the transition to school to subsequent teacher ratings of internalizing and externalizing behaviors (Hughes and Ensor, 2011). Additional research is needed to explore how early manifestations of trait anxiety impair attentional control and thus affect executive functioning across neutral or emotionally-salient contexts, and how, in turn, reduced cognitive control further amplifies the information processing patterns specific to anxious cognition and behavior (e.g., Pine, 2007).

### Current study

EF dimensions have been shown to undergo intensive developments between the ages of 3 and 6, and their progress during this sensitive developmental window predicted a wide range of cognitive, emotional, and educational outcomes. However, the dependency of these distal outcomes on more basic attentional /memory prerequisites across the preschool years has been theoretically postulated (e.g., Garon et al., 2008), yet not empirically documented. Also, reciprocal links between individual differences in anxiety and various EF dimensions during the preschool years and the transition to school have been identified. However, their interplay has not been systematically investigated. Consequently, the current study was designed to address these two key research questions regarding the developmental EF precursors and early links to individual differences in anxiety, both viewed through the lenses of their early reliance on attentional control mechanisms. Several secondary questions were addressed along the way.

A first aim was to investigate whether EF outcomes (WM, inhibition, shifting) measured during late preschool years could be predicted by children's earlier (9 months) assessed STM spans. We expected greater coherence between measures of verbal and visuospatial WM and their respective STM predictors (Alloway et al., 2006). We also attempted to confirm findings by Espy and Bull (2005), who related measures of response conflict, but not of response suppression, to children's memory spans. To our knowledge, this is the first time that children's performance on a shifting task was related to previous and concurrent levels of STM functioning. A secondary aim was related to the development of STM itself during the preschool years, across the verbal and visuospatial domains. This complements the limited body of longitudinal data documenting intensive progress in children's memory span during this interval (e.g., Gathercole et al., 1992; Schneider et al., 2004; Visu-Petra et al., 2009). The cross-sectional progress for all our measures was followed in order to check for performance improvements in children between 3 and 6 years old.

The second aim concerned the role of individual differences in children's EF performances. In this respect, anxiety-related worrisome thoughts are presumed to generate a cognitive interference, mostly visible in tasks high on executive-demands and/or manipulating verbal information (Eysenck et al., 2007). Hence, we hypothesized that higher levels of anxiety would be related to performance deficits on executive-demanding tasks (especially on verbal WM, response conflict, and set-shifting measures), and to a lesser degree on tasks involving lower executive demands (STM and response suppression). We investigated the role of such individual differences in anxiety while controlling for other individual differences variables such as non-verbal intelligence, age, or gender. Most of our tasks, with the exception of the Em-DCCS, did not require children to process emotional information. Previous studies conducted in the ACT (Eysenck et al., 2007) framework indicate that even in such neutral contexts, especially in high executive-demanding ones, anxiety-related performance deficits can be evident. To our knowledge, this is the first study to systematically link early individual differences in anxiety symptoms to subsequent EF performance.

## Materials and methods

### Participants and procedure

The initial sample consisted of 76 preschoolers recruited from three public kindergartens in the northwest of Romania. However, 8 children could not be followed-up at the second time point (T2), hence data from a total of 68 preschool children (41 boys), aged between 3 years and 2 months and 6 years and 8 months (*M* = 4 years and 8 months, *SD* = 10.5 months) at the first assessment (T1), are presented in the current study. Parents who approved their children's participation were also asked to complete a form requiring demographic information, with exclusion criteria such as neurological or psychological disorders. Aside from parental written consent, the child's verbal assent was also obtained prior to testing. All participants were monolingual Romanian-speaking children, living in urban areas.

Children of parents who gave their written consent were tested individually in a quiet room located at their kindergartens. At T1, all preschoolers were tested in a single session with measures of non-verbal intelligence (Colored Progressive Matrices test), verbal STM (Digit Span and Word Span) and visuospatial STM (Corsi blocks test). Nine months later (at T2), tasks were administered in three separate sessions in order to avoid preschoolers' fatigue and boredom. Hence, in the first session at T2, children were evaluated with the same STM tests administered at T1, with an additional Articulation Rate task, which is not described in this study. Verbal WM (Counting, Backwards Digit, and Listening span) and visuospatial WM tasks (Mr. X, Odd-one-out) were administered in the second session. Finally, inhibition and set-shifting performance were evaluated during a third session (Statue, Knock and Tap, Day/Night Stroop, Em-DCCS), in order to minimize fatigue effects.

### Measures

#### Individual differences in intelligence and anxiety

*Non-verbal intelligence* was assessed using the ***Colored Progressive Matrices test*** (Raven et al., 1998) designed to be suitable for young children. This test consists in 36 individual patterns, for each of which children have to correctly identify the missing segment (out of 6 possible segments). The total number of correct responses provides a non-verbal intelligence measure for each child.

*Trait anxiety* was evaluated via parental report on the ***Spence Preschool Anxiety Scale*** (Spence et al., 2001; the Romanian version Benga et al., 2010). The scale consists of 28 anxiety items, 5 non-scored posttraumatic stress disorder items, and another open-ended (non-scored) item. Each parent rated the concordance between the child's behavior and the one described in each item on a 5 point scale. Parents' ratings of the children's anxiety symptoms generated a total score which provided an overall measure of each child's trait anxiety. The trait anxiety measure was administered at T1 only.

#### Short term memory

During the ***Digit Span*** task (Forward subtest, WISC-III; Wechsler, 1991), children were instructed to repeat each digit sequence spoken by the experimenter in the correct order. The test consists of 9 blocks of 3 trials each. Trials of 2 digits each are included in the first block, after which STM span requirements gradually increase to trials of 9 digits each in the last block. If children correctly recall two trials in a block, the experimenter increases span requirements by moving on to the next block. If the child fails two trials in a block, testing is discontinued.

For the ***Word Span*** task, a list of 9 common two-syllable words was chosen to provide a test of word repetition directly comparable to the other span measures. Two-syllable words were chosen in order to avoid possible word length effects, and to provide a measure more directly comparable to the word length of items from digit span (in Romanian, five out of nine digits have two syllables). Besides stimulus type (words), the task was identical to the Digit Span task in all respects.

Visuospatial STM was evaluated using the ***Corsi blocks test*** (Corsi, 1972). For this test, we used the display provided by the WAIS-R Neuropsychological Inventory (Kaplan et al., 1991). Children were presented with 10 blue cubes randomly located on a board. During task unfolding, the examiner taps a sequence of cubes, and the child is required to reproduce the sequence, by tapping the cubes in the correct order. Besides stimulus type (cube locations), the task was identical to the Digit and Word Span.

***STM scoring***. Aggregate scores for STM spans were computed following the procedure described by Cowan and collaborators (2003). First, the base span, the highest list length at which the responses for all sequences were correct, was extracted, and a score of 0.33 was added for every correct sequence above this base span. Additionally, a general index of verbal STM was computed by averaging the Word and Digit aggregate spans.

#### Working memory

WM was evaluated using tasks from the Automated Working Memory Assessment battery (AWMA; Alloway, 2007), a widely-used measure for WM assessment in 4- to 11-year-old children. Three measures were administered in order to assess verbal WM (Counting Recall, Backwards digit recall, Listening recall), while two other (Odd-one-out and Mr. X) were employed to evaluate visuospatial WM. In all these tasks, a particular list length contains 6 trials—if the child correctly performs 3 trials from a list length, the program automatically skips to the next list length. If less than 3 trials from a list length are correctly recalled, testing stops for that task.

In the ***Counting Recall*** test, children are presented with a visual array of red circles and blue triangles. They are asked to count the number of circles in each array, and to memorize the totals. At the end of each trial, children are required to recall the number of circles included in each array, in the correct order. The test consists of 7 blocks of 6 trials each, beginning with trials of one array in the first block, increasing to trials of 7 arrays in the last block.

The ***Backwards Digit Recall*** test is identical to the Digit Span task, except children are required to recall a gradually increasing sequence of spoken digits in the reversed order. The sequences increase by one digit from one block to another, with a maximum of 7 digits for trials corresponding to the last block. The ***Listening Recall*** task consists in a series of short sentences (e.g., “The grass is blue” and “Sugar is sweet”) for which children are asked to judge the veracity by giving a “yes” or “no” response to the experimenter. After judging the veracity of each sentence in a trial, children are required to recall the final word of each sentence within the given trial (e.g., “blue” and “sweet”). The test consists of 6 blocks of 6 trials each, with the number of sentences within each trial gradually increasing from two to six.

In the ***Odd-one-out*** task (adapted by the AWMA authors from Russell et al., 1996) children are presented with three shapes, each in a box, displayed in a row. They are then asked to point the odd shape out of each row. After this, the shapes disappear and the child is presented with three empty boxes, being asked to point to where the odd shape was. From the initial level presenting only one row of shapes, difficulty increases up to 7 rows, children being asked to recall the location of the odd shape from each row, in the order they had been shown in each trial. In the ***Mr. X*** task, two fictitious cartoon figures, presented as “Mr. X with the blue hat” and “Mr. X with the yellow hat,” are displayed in each item. Children are first asked to identify whether Mr. X with the blue hat is holding *a ball* in the same hand as Mr. X with the yellow hat. With span requirements increasing, more Mr. Xs appear on each block and the child is asked to recall the location of each ball by pointing to a picture with eight compass points. The task consists of 7 blocks of 6 trials each, location span gradually increasing by one with each block.

***WM scoring***. Aggregate WM spans were computed in the same manner as aggregate STM spans, except that this time a 0.17 score was added to the base span, as one level consisted of 6 trials. Verbal WM and visuospatial WM composite scores were calculated by averaging the scores on corresponding verbal and visuospatial tasks.

#### Inhibition

In order to assess Inhibition, we used a task requiring simple response suppression, as well as two tasks generating response conflict.

***The Statue*** task from the NEPSY-I battery (Korkman et al., 1998) evaluates *response suppression*, requiring motor persistence when several distracters are introduced. Children are required to stand in a “statue” position, refraining from vocalizations and body movements for 75-s. During this interval, pre-set distracters are introduced (the examiner coughing, dropping a pen etc.). A 2 points score is attributed for inhibiting any response over each 5-s interval, and a 1 point score for displaying one inappropriate response. The maximum score to be earned by not doing anything throughout this interval is 30.

***Knock and Tap*** is a classical non-verbal Go/No-Go task included in the NEPSY-I battery (Korkman et al., 1998), evaluating *motor response conflict* between immediate motor responses triggered by visual stimuli and the action that is specified in previous verbal directions (Klenberg et al., 2001). In the first part of the test (Part A), children are asked to knock on the table when the examiner taps and vice-versa during 15 trials. In the second part of the task (Part B), children are required to shift to a new set of response. Specifically, they are taught to tap with the side of their first when the examiner knocks and vice-versa, but also to inhibit any motor response when the examiner taps. Part B also consists of 15 trials, and the total number of correct responses (out of 30) determines the accuracy score.

The version of ***Day/Night Stroop*** that we used is an uninterrupted measure of *verbal response conflict*, in which children are presented with a matrix displaying 16 pictures of the sun and moon, respectively. Participants were asked to name the pictures from left to right on each of the four rows, but to inhibit their prepotent responses and say “night” when pointing to the sun, and “day” when pointing to the picture depicting the moon. Thus, we transformed the standard version of the task (Gerstadt et al., 1994) into a more self-paced, speeded task. The maximum accuracy score was 16, and the experimenter timed children's total response in order to obtain an efficiency measure. Accuracy scores may be sufficient for measuring young children's inhibition (e.g., Diamond and Kirkham, 2001), yet in school age children and adults, measures of response time proved to be more sensitive measures, especially when accuracy performance points toward ceiling effects (e.g., MacLeod, 1991; Wright et al., 2003). This later approach was also successfully used with children as young as 3½ years (Simpson and Riggs, 2005). Hence, both latency and accuracy of response were taken into account to generate an inverse-efficiency score (Kennett et al., 2001), calculated as total response time divided by the proportion of correct responses for each participant. Lower values on this measure indicate better inhibitory performance.

#### Shifting

**Figure 1:**
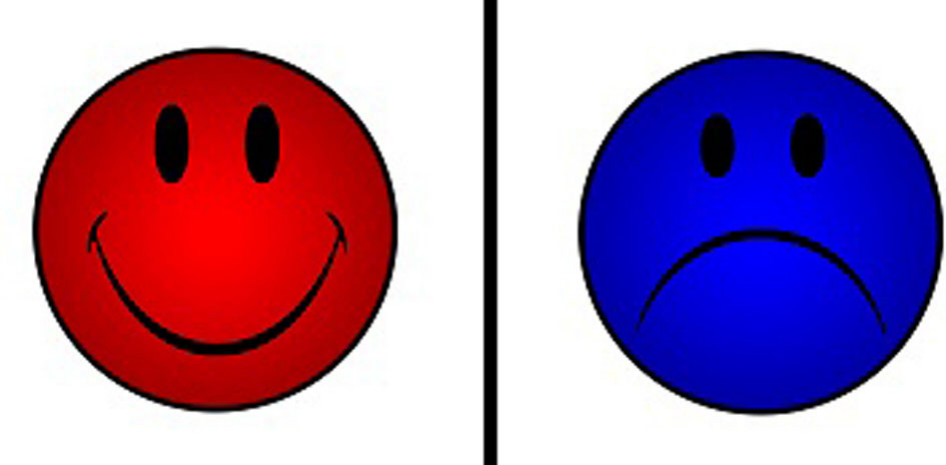
**Target cards for the EM-DCCS task**.

Finally, shifting performance was estimated using the ***Emotional- Dimensional Change Card Sort (Em-DCCS)***. The classic DCCS task provides a measure of cognitive flexibility in children as young as 3 (Zelazo, 2006). In the emotional version of the task, the target cards consisted of a happy red face and a sad blue face, and their placing (left or right) was counterbalanced across the sample. The version used in this study was modified by using schematic emotional faces, as they were successfully used in previous research regarding anxiety-related bias effects in children (e.g., Hadwin et al., 2003). The schematic faces are presented in Figure 1.

Participants were initially requested to sort the six pre-switch test cards by the color criterion. After the six pre-switch trials, the experimenter said: “*Now we are going to play another game. We are not going to play the color game anymore. We are going to play the faces game.*” Only performance on the post-switch trials was analyzed, after data from one child who scored poorly (less than 5 out of 6) in the pre-switch phase were excluded. Due to the non-normal distribution of scores on the post-switch phase and the overall high levels of performance, performance was dichotomized using a more stringent criterion than for the pre-switch phase. Thus, two groups were created, children who could perfectly switch to the emotional judgment on all trials of the post-switch phase (*n* = 34) and children with less than perfect performance (*n* = 33).

## Results

*Analytic approach*. In order to determine whether performance on STM tasks was associated with children's EF performance 9 months later, beyond other first assessment measures, separate hierarchical regressions were carried out for each EF outcome (verbal and visuospatial WM, response suppression, verbal and motor response conflict, and attention shifting). The association between individual differences in non-verbal intelligence and trait anxiety was tested in the same manner, after first controlling for the age and gender of the participants. We further tested whether concurrent levels of STM were useful in the prediction of EF outcomes beyond the first assessment STM, age, gender, non-verbal intelligence, and anxiety.

### Preliminary analyses

**Figure 2:**
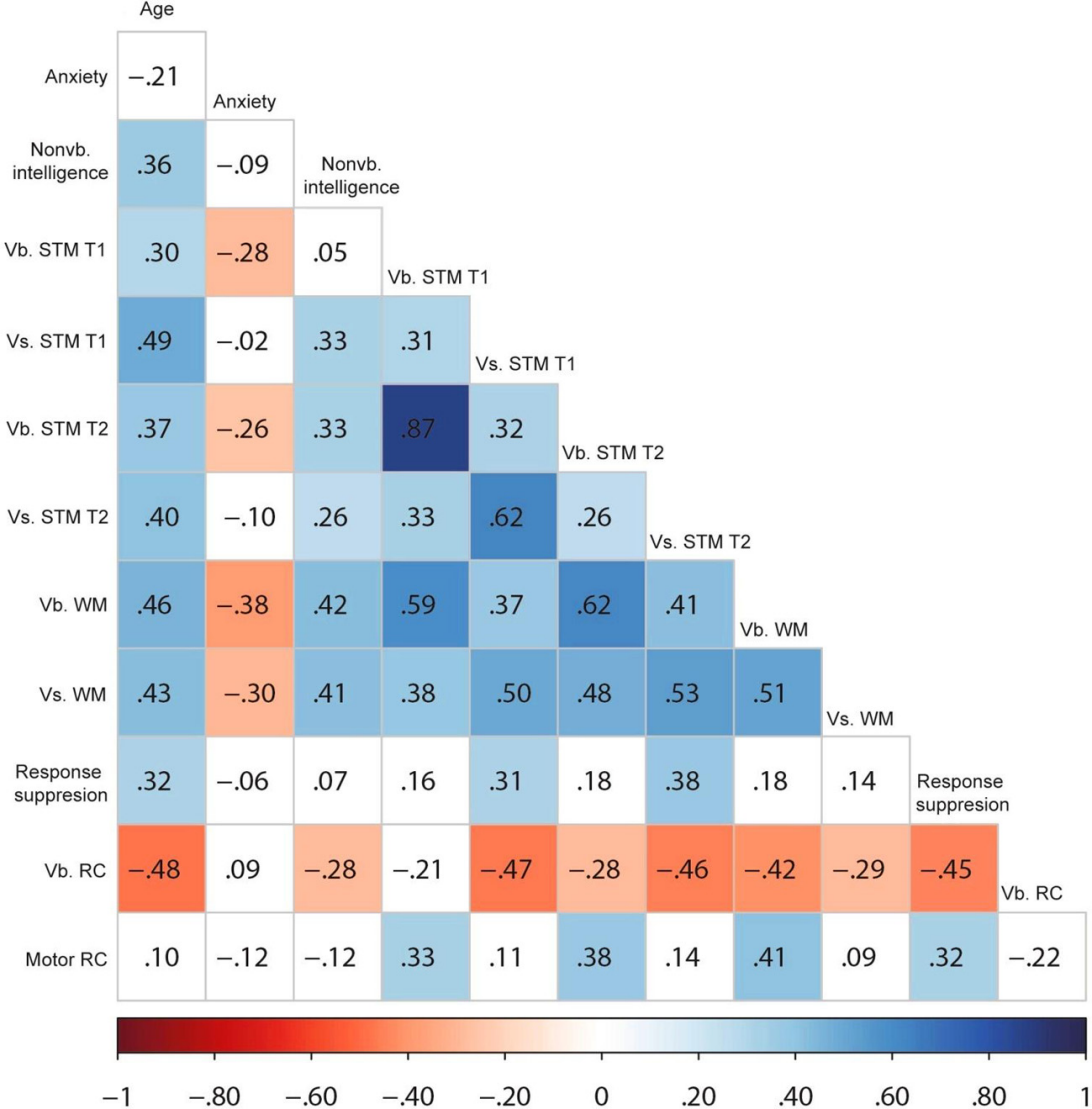
**Pearson bivariate correlation matrix for the measures of interest**. Color saturation represents the correlation strength according to the scale below the figure. Vb., verbal; Vs., visuospatial; STM, short-term memory; WM, working memory; RC, Response conflict.

During the univariate and bivariate graphical examination of data, three outlying observations were identified and discarded as they were situated more than 3 SDs below/above the sample means (two on the Day/Night Stroop matrix and one on the Knock and Tap task). One child with poor performance on the pre-switch phase (2/6) of the DCCS task was excluded from the shifting analysis. Univariate descriptives on all measures are listed in Table 1, and Figure 2 presents associations between measures of interest. The correlation matrix for all recorded measures is presented in the Supplementary Materials.

**Table 1:** **Univariate descriptives for the raw and composite scores**.

**Type of measure**	**Tasks**	***N***	**Mean**	***SD***	**Median**	**Min**	**Max**	**Skew**	**Kurt**.
**TIME 1**									
Age (in months)	–	68	56.00	10.54	56.0	38.00	80.00	0.12	−0.90
Nonvb. intelligence	Raven	68	16.00	4.05	15.50	8.00	27.00	0.59	0.09
Trait anxiety	Spence	68	28.50	14.36	25.0	6.00	68.00	0.55	−0.42
Verbal STM	Word span	66	3.60	0.74	3.70	2.00	5.30	0.02	−0.17
	Digit span	66	4.00	0.84	4.00	2.00	6.30	0.31	0.53
Visuospatial STM	Corsi blocks	68	2.80	0.66	2.70	1.66	5.00	0.81	0.92
**TIME 2**									
Verbal STM	Word span	68	4.00	0.63	4.00	2.33	5.30	−0.21	−0.28
	Digit span	68	4.20	0.83	4.00	2.33	6.30	0.35	−0.32
Visuospatial STM	Corsi blocks	68	2.90	0.70	2.70	1.33	5.00	0.47	0.17
Verbal WM	Counting span	63	1.62	0.58	1.50	1.00	3.00	0.54	−0.75
	Backward span	63	1.11	0.59	1.00	0.17	2.70	0.19	−0.25
	Listening span	61	1.23	0.67	1.33	0.33	2.30	−0.44	−1.44
Visuospatial WM	Mr. X	63	0.84	0.53	0.84	0.17	1.70	0.27	−1.32
	Odd-one-out	63	1.61	0.51	1.50	0.67	2.70	0.15	−0.77
Response suppression	Statue	68	25.65	4.80	27.00	10.00	30.00	−1.77	2.58
Response conflict	Stroop matrix accuracy	68	13.94	2.97	15.00	2.00	16.00	−1.86	3.77
	Stroop matrix IE	66	1.61	0.77	1.40	0.69	4.20	1.44	2.01
	Knock and Tap	67	27.21	2.78	28.00	18.00	30.00	−1.31	1.46
Shifting	Post-switch DCCS	67	4.24	1.94	6	1	6	−0.33	−1.70
Composite measures	Vb. WM	61	1.32	0.52	1.39	0.50	2.28	0.08	−1.02
	Vs. WM	63	1.23	0.46	1.17	0.42	2.17	0.30	−0.88

Inverse efficiency (IE) was calculated as response time divided by accuracy. Kurt., Kurtosis.

#### Associations between measures at T1

Older children presented higher non-verbal intelligence (Raven) scores, *r*_(66)_ = 0.36, *p* = 0.01, as well as superior verbal STM, *r*_(64)_ = 0.30, *p* = 0.02, and visuospatial STM spans, *r*_(64)_ = 0.49, *p* < 0.001. Non-verbal intelligence was significantly associated with visuospatial STM, *r*_(64)_ = 0.33, *p* < 0.001, but not with verbal STM, *r*_(64)_ = 0.05, *p* < 0.71. On the other hand, trait anxiety (Spence Preschool Anxiety Scale) negatively correlated with verbal STM span, *r*_(64)_ = −0.28, *p* = 0.02, yet was not associated with visuospatial STM, *r*_(64)_ = −0.02, *p* = 0.89. The results also revealed a significant association between verbal and visuospatial STM composite scales, *r*_(64)_ = 0.31, *p* = 0.01. There were no gender-related differences regarding non-verbal intelligence, anxiety, and STM.

#### Associations between measures at T2

At T2, there was a again a significant association between verbal and visuospatial STM spans, *r*_(66)_ = 0.26, *p* = 0.03. There was also a positive correlation between verbal and visuospatial WM, *r*_(61)_ = 0.51, *p* < 0.001. A test for the equality of correlations (using the Fisher z transformation) revealed that the correlation between the verbal and visuospatial scales was significantly stronger for WM than for STM, *z* = 1.67, *p* = 0.05, 1-tailed. As expected, verbal STM at T2 correlated positively with verbal WM, *r*_(61)_ = 0.62, *p* < 0.001, while visuospatial STM at T2 was positively associated with visuospatial WM, *r*_(61)_ = 0.53, *p* < 0.001.

The pattern of results regards correlations between WM composite spans and inhibition measures was mixed. Verbal WM correlated positively with motor response conflict (Knock and Tap), *r*_(60)_ = 0.41, *p* = 0.01, and negatively with the (response time based) measure of verbal response conflict (Day/Night Stroop), *r*_(59)_ = −0.42, *p* < 0.001, but did not correlate with response suppression (Statue), *r*_(61)_ = 0.18, *p* = 0.17. Similarly, verbal STM (at T2) correlated positively with motor response conflict (Knock and Tap), *r*_(65)_ = 0.38, *p* = 0.01, and negatively with verbal response conflict (Day/Night Stroop), *r*_(64)_ = −0.28, *p* = 0.02, but did not correlate with response suppression (Statue), *r*_(66)_ = 0.18, *p* = 0.14. The only inhibition measure associated with visuospatial WM was verbal response conflict (Day/Night Stroop), *r*_(59)_ = −0.29, *p* = 0.02. Visuospatial STM (at T2) correlated significantly with both verbal response conflict (Day/Night Stroop), *r*_(64)_ = −0.46, *p* = 0.01, and response suppression (Statue), *r*_(66)_ = 0.38, *p* = 0.01. The correlation between motor (Knock and Tap) and verbal (Day/Night Stroop) response conflict was non-significant, *r*_(64)_ = −0.22, *p* = 0.07. However, response suppression (Statue) correlated with both motor, *r*_(65)_ = 0.32, *p* = 0.01, and verbal response conflict, *r*_(64)_ = −0.45, *p* < 0.001.

#### Longitudinal associations

The associations between STM spans at the two time points were substantial, particularly for verbal tasks, *r*_(64)_ = 0.87, *p* < 0.001. The gains in STM (calculated as the difference between T2 and T1 spans) correlated significantly and negatively with the corresponding STM spans at T1, *r*_(64)_ = −0.48, *p* < 0.001, for verbal STM, and *r*_(66)_ = −0.37, *p* = 0.01, for visuospatial STM. A paired *t*-test revealed that gains in verbal STM were highly significant, as the difference between the two time points was, on average 0.26 (95% CI from 0.17 to 0.35). The visuospatial STM gains were also significant, with a mean difference of 0.14 (95% CI from 0.01 to 0.29), but the estimate of the mean difference lacked precision due to the large variance in gains (*SD* = 0.59). The results revealed no significant links between the STM gains and anxiety, or non-verbal intelligence. STM spans at T1 correlated moderately with the corresponding WM spans, *r*_(59)_ = 0.59, *p* < 0.001, for verbal measures, and *r*_(61)_ = 0.50, *p* < 0.001, for visuospatial measures.

With regards to associations with the individual differences measured at T1, results revealed that non-verbal intelligence was positively associated to verbal WM scores, *r*_(61)_ = 0.42, *p* < 0.001, and visuospatial WM, *r*_(61)_ = 0.41, *p* < 0.001. The only other EF measure associated with non-verbal intelligence was the Day/Night Stroop inverse efficiency, *r*_(64)_ = −0.28, *p* = 0.02, revealing that children with higher non-verbal intelligence scores also had superior performances in terms of verbal response conflict (Day/Night Stroop). At the same time, correlations also revealed that higher anxiety was linked to lower verbal STM spans at T2, *r*_(66)_ = −0.26, *p* = 0.04, as well as to lower verbal and visuospatial WM spans, −0.38 < *r* < −0.30. However, trait anxiety was not significantly related to response conflict (Knock and Tap, and Day/Night Stroop) or response suppression (Statue). The mean anxiety score of children who did not pass the shifting task (DCCS, *M* = 32.20, *SD* = 12.10) was significantly higher than that of the children who passed (*M* = 24.79, *SD* = 15.85), *t*_(60)_ = 2.17, *p* = 0.03. The only T2 measure for which gender effects were found was attention shifting as the odds of maximal performance for girls were 6.11 times (95% CI from 1.99 to 18.76) the odds of boys.

### Cross-sectional effects of age

**Figure 3:**
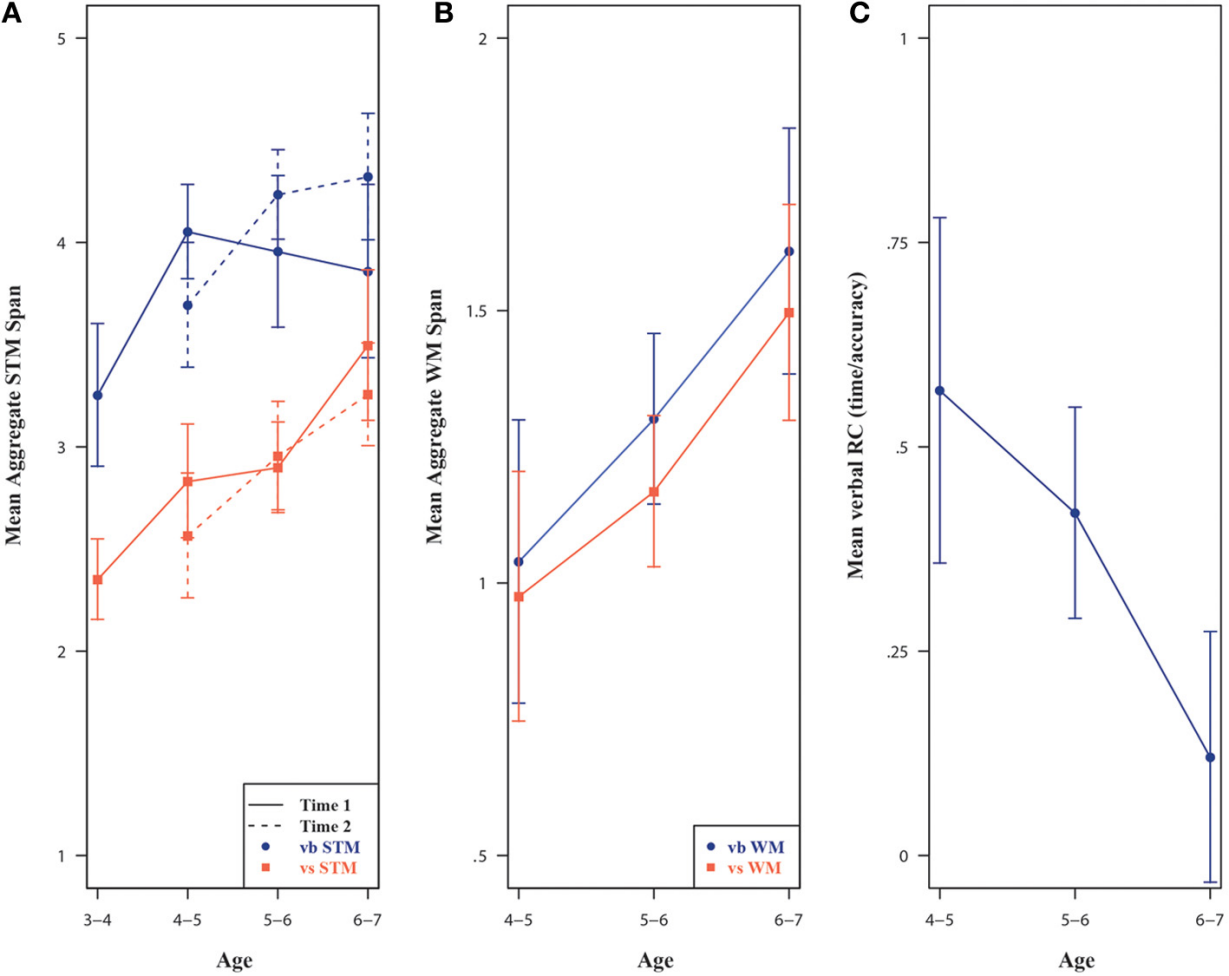
**Children's mean STM (A), WM (B), and verbal response conflict (C) performances with 95% Confidence Intervals**. Performance for verbal response conflict was assessed using the log inverse efficiency score (total time to complete the task/total score). Vb., verbal; Vs., visuospatial; STM, short-term memory; WM, working memory; RC, Response conflict.

The current section charts the age-related progress in both STM and EF abilities through a descriptive, cross-sectional approach. The graphical exploration in Figure 3A suggests that the most substantial improvements in terms of verbal STM span roughly occurred between the age of 3–4 to 4–5 years, after which performance stagnated or had a more modest increase up to the age of 6–7 years. The only significant increase in verbal STM performance at T1 was evident when comparing 3- (*M* = 3.25, *SD* = 0.74) to 4-year-olds (*M* = 4.05, *SD* = 0.62), *t*_(30)_ = 3.72, *p* < 0.001. However, at T2, 5-year-olds (*M* = 4.23, *SD* = 0.62) significantly outperformed 4-year-olds (*M* = 3.69, *SD* = 0.64), *t*_(32)_ = 2.83, *p* = 0.01. This discrepancy made it difficult to pinpoint the exact age at which peak performance in verbal STM was achieved. However, it is certain that 6-year-olds did not outperform 5-year-olds in terms of verbal STM at any time point.

For visuospatial STM (see Figure 3A), children's improvement was more gradual and continuous across the whole age range. Based on the T2 assessment of their visuospatial STM span performance, the difference in estimated means between children 1-year apart was of 0.32 (95% CI from 0.16 to 0.48).

The improvements in WM spans (see Figure 3B) followed a fairly linear trend, although there was considerable variability in performance within each 1 year age range (*SDs* = 0.40). Older children had mean verbal WM spans 0.27 higher (95% CI from 0.14 to 0.40) than their 1 year younger peers. Older children also had a 0.23 (95% CI from 0.11 to 0.34) increase in estimated mean visuospatial WM span.

Similarly, the response time-based measure of verbal response conflict (i.e., log Day/Night Stroop inverse efficiency) followed a downward linear trend as age increased (see Figure 3C). A one-year increase in age was related to a change in estimated mean Day/Night Stroop performance of −0.39 (95% CI from −0.59 to −0.20). However, older children did not outperform their younger peers on motor response conflict (Knock and Tap) as a 1-year increase in age was associated with a 0.13 increase in mean motor response conflict (95% CI from −0.25 to 0.52). On the response suppression task, a 1-year increase in age was associated with an increase of 1.73 (95% CI from 0.48 to 2.98) in estimated mean response suppression (Statue). The percentage of children passing on the post-shift DCCS increased from 25% (for 4-year olds) to 67% (for 6-year olds).

### Concurrent and predictive effects

Separate eight step hierarchical regressions were conducted for each outcome (verbal and visuospatial WM, response suppression, verbal and motor response conflict, and shifting). For all outcomes, the first four predictors included in the regressions were: age at T1 (step 1), gender (step 2), non-verbal intelligence (step 3), and trait anxiety (step 4). In step 5, verbal STM at T1 was added as a predictor of verbal WM, and visuospatial STM at T1 as a predictor of visuospatial WM. In the subsequent steps, we added the domain non-specific STM measures at T1 (i.e., verbal STM for visuospatial WM, and visuospatial STM for verbal WM; step 6), followed by the domain specific STM measures at T2 (step 7) and the domain non-specific STM measures at T2 (step 8). For all other EF outcomes, the remaining predictors were added as follows: verbal STM at T1 (step 5), visuospatial STM at T1 (step 6), verbal STM at T2 (step 7), visuospatial STM at T2 (step 8). Hierarchical regression models with the coefficient of determination (*R*^2^) at each step and the *F*-tests comparing consecutive models are presented in Table 2 for WM, as well as in Table 3 for inhibition and shifting.

**Table 2:** **Hierarchical regression models predicting children's performance on verbal and visuospatial working memory (WM) spans**.

	**Verbal WM**			**Visual WM**		
	***B***	***SE***	***β***	***B***	***SE***	***β***
**STEP 1**						
Intercept	0.03	0.33	–	0.15	0.29	–
Age	0.02	0.01	0.45^***^	0.02	0.00	0.43^***^
*R*^2^(Δ*F*)	0.21 (15.31^***^)			0.19 (13.50^***^)		
**STEP 2**						
Intercept	−0.004	0.33		0.15	0.30	
Age	0.02	0.01	0.32^***^	0.02	0.00	0.43^***^
Gender	0.17	0.12	0.16	−0.01	0.11	−0.01
*R*^2^(Δ*F*)	0.23(1.96)				0.19 (0.01)	
**STEP 3**						
Intercept	−0.26	0.34	–	−0.02	0.31	–
Age	0.02	0.01	0.32^*^	0.01	0.01	0.34^*^
Gender	0.20	0.12	0.19	0.01	0.11	0.01
Nonvb. Intelligence	0.04	0.02	0.28^*^	0.03	0.02	0.22
*R*^2^(Δ*F*)	0.30 (5.32^*^)			0.23 (2.91)		
**STEP 4**						
Intercept	0.32	0.38	–	0.38	0.36	–
Age	0.01	0.01	0.24^*^	0.01	0.01	0.28^*^
Gender	0.21	0.11	0.19	0.01	0.10	0.01
Nonvb. Intelligence	0.03	0.02	0.25	0.02	0.01	0.20
Anxiety	−0.01	0.00	−0.31^**^	−0.01	0.00	−0.25^*^
*R*^2^(Δ*F*)	0.39 (8.08^**^)			0.28 (4.44^*^)		
**STEP 5**						
Intercept	−0.83	0.40	–	0.15	0.35	–
Age	0.01	0.00	0.16	0.00	0.01	0.11
Gender	0.17	0.09	0.16	0.00	0.01	0.00
Nonvb. Intelligence	0.04	0.01	0.28^**^	0.02	0.01	0.17
Anxiety	−0.01	0.00	−0.19^+^	−0.01	0.00	−0.26^*^
Domain specific STM (T1)	0.32	0.07	0.46^***^	0.25	0.09	0.35^**^
*R*^2^(Δ*F*)	0.57 (23.18^***^)			0.37 (7.98^**^)		
**STEP 6**						
Intercept	−0.86	0.40	–	−0.25	0.41	–
Age	0.01	0.01	0.12	0.00	0.01	0.09
Gender	0.17	0.09	0.16	−0.01	0.01	−0.01
Nonvb. Intelligence	0.04	0.01	0.27^**^	0.02	0.01	0.19
Anxiety	−0.01	0.00	−0.19^*^	−0.01	0.00	−0.20
Domain specific STM (T1)	0.31	0.07	0.44^***^	0.22	0.09	0.30^*^
Domain non-specific STM (T1)	0.07	0.09	0.09	0.12	0.07	0.20
*R*^2^(Δ*F*)	0.57 (0.69)			0.41 (2.99)		
**STEP 7**						
Intercept	−0.95	0.44	–	−0.28	0.40	–
Age	0.00	0.01	0.11	0.00	0.01	0.09
Gender	0.17	0.10	0.16	−0.01	0.09	−0.01
Nonvb. Intelligence	0.03	0.01	0.26^*^	0.02	0.01	0.15
Anxiety	−0.01	0.00	−0.18	−0.01	0.00	−0.20
Domain specific STM (T1)	0.24	0.14	0.34	0.10	0.10	0.14
Domain non-specific STM (T1)	0.08	0.09	0.10	0.09	0.07	0.15
Domain specific STM (T2)	0.09	0.17	0.11	0.18	0.08	0.30^*^
*R*^2^(Δ*F*)	0.58 (0.29)			0.46 (4.89^**^)		
**STEP 8**						
Intercept	−0.99	0.44	–	−0.48	0.43	–
Age	0.00	0.01	0.10	0.00	0.01	0.06
Gender	0.18	0.10	0.17	0.01	0.01	0.01
Nonvb. Intelligence	0.03	0.01	0.24^*^	0.01	0.01	0.10
Anxiety	−0.01	0.00	−0.18	−0.00	0.00	−0.18
Domain specific STM (T1)	0.21	0.14	0.30	0.11	0.10	0.16
Domain non-specific STM (T1)	0.03	0.10	0.04	−0.06	0.14	−0.10
Domain specific STM (T2)	0.12	0.17	0.14	0.20	0.08	0.32^*^
Domain non-specific STM (T2)	0.08	0.09	0.12	0.20	0.17	0.29
*R*^2^(Δ*F*)	0.58 (0.98)			0.47 (1.46)		

β, Standardized regression coefficient; T1, first time assessment; T2, second time assessment; Domain specific STM, verbal STM for verbal WM, and visuospatial STM for visuospatial WM. Domain non-specific STM, visuospatial STM for verbal WM, and verbal STM for visuospatial WM. The baseline gender is male.

+ *p < 0.06*,

* *p < 0.05*,

** *p < 0.01*,

*** p < 0.001.

**Table 3:** **Hierarchical regression models predicting children's performance on inhibition (verbal and motor response conflict, and response suppression) and shifting measures**.

	**Verbal RC**			**Motor RC**			**Response suppression**			**Shifting**		
	***B***	***SE***	***β***	***B***	***SE***	***β***	***B***	***SE***	***β***	***B***	***SE***	***β***
**STEP 1**												
Intercept	1.52	0.26		25.64	1.90		17.33	3.07		−3.67	1.50	
Age	−0.02	0.00	−0.49^***^	0.03	0.03	0.10	0.15	0.05	0.33^**^	0.07	0.03	0.68^*^
*R*^2^(Δ*F*)	0.24 (19.80^***^)			0.01 (0.66)			0.11 (7.64^**^)			0.10 (6.80^**^)		
**STEP 2**												
Intercept	1.52	0.26		25.58	1.92		17.20	3.10		−2.83	1.64	
Age	−0.02	0.00	−0.49^***^	0.03	0.03	0.10	0.15	0.05	0.32^**^	0.07	0.03	0.77^*^
Gender	0.01	0.10	0.02	0.17	0.72	0.03	0.51	1.17	0.05	−2.02	0.63	−2.02^***^
*R*^2^(Δ*F*)	0.24 (0.02)			0.01 (0.06)			0.11 (0.19)			0.25 (12.15^***^)		
**STEP 3**												
Intercept	1.62	0.29		27.20	2.06		17.65	3.43		−4.17	1.91	
Age	−0.02	0.00	−0.46^***^	0.05	0.03	0.20	0.15	0.06	0.34^*^	0.06	0.03	0.59
Gender	0.01	0.10	0.01	0.05	0.70	0.01	0.47	1.19	0.05	−2.23	0.66	−2.23^***^
Nonvb. intelligence	−0.01	0.01	−0.10	−0.19	0.10	−0.26	−0.05	0.17	−0.04	0.15	0.09	0.62
*R*^2^(Δ*F*)	0.25 (0.73)			0.07 (3.81^+^)			0.11 (0.10)			0.29 (3.13)		
**STEP 4**												
Intercept	1.62	0.34		28.89	2.36		17.49	4.05		−2.18	2.13	
Age	−0.02	0.00	−0.46^***^	0.05	0.03	0.17	0.16	0.06	0.34^*^	0.05	0.03	0.56
Gender	0.01	0.10	0.01	0.07	0.70	0.01	0.47	1.20	0.05	−2.43	0.71	−2.43^***^
Nonvb. intelligence	−0.01	0.01	−0.10	−0.21	0.10	−0.29^*^	−0.05	0.17	−0.04	0.14	0.09	0.55
Anxiety	0.00	0.00	−0.00	−0.04	0.02	−0.18	0.00	0.04	0.01	−0.05	0.02	−0.78^*^
*R*^2^(Δ*F*)	0.25 (0.00)			0.10 (2.05)			0.11 (0.01)			0.34 (5.25^*^)		
**STEP 5**												
Intercept	1.72	0.41		25.36	2.78		16.01	4.98		−6.04	3.01	
Age	−0.02	0.00	−0.44^***^	0.02	0.04	0.09	0.15	0.06	0.32^*^	0.03	0.04	0.35
Gender	0.01	0.10	0.01	−0.08	0.68	−0.01	0.42	1.21	0.04	−2.47	0.75	−2.47^***^
Nonvb. intelligence	−0.01	0.01	−0.11	−0.19	0.10	−0.26	−0.04	0.17	−0.03	0.18	0.10	0.72
Anxiety	0.00	0.00	−0.02	−0.02	0.02	−0.11	0.01	0.04	0.03	−0.05	0.03	−0.66
Vb. STM (T1)	−0.03	0.07	−0.06	1.07	0.48	0.29^*^	0.46	0.85	0.07	1.08	0.51	0.81
*R*^2^(Δ*F*)	0.25 (0.21)			0.17 (4.94^*^)			0.11 (0.27)			0.39 (5.25^*^)		
**STEP 6**												
Intercept	1.77	0.40		25.37	2.81		15.56	4.95		−6.08	3.01	
Age	−0.01	0.01	−0.32^*^	0.02	0.04	0.10	0.10	0.07	0.23	0.03	0.04	0.36
Gender	0.01	0.10	0.01	−0.08	0.68	−0.01	0.41	1.20	0.04	−2.46	0.75	−2.46^***^
Nonvb. intelligence	−0.01	0.01	−0.08	−0.19	0.10	−0.26	−0.06	0.17	−0.05	0.18	0.10	0.72
Anxiety	0.00	0.00	0.01	−0.02	0.03	−0.11	0.00	0.00	0.01	−0.05	0.03	−0.65
Vb. STM (T1)	−0.01	0.07	−0.02	1.07	0.49	0.29^*^	0.12	0.87	0.03	1.09	0.53	0.82^*^
Vs. STM (T1)	−0.17	0.09	−0.25	−0.05	0.65	−0.01	1.53	1.13	0.20	−0.04	0.61	−0.03
*R*^2^(Δ*F*)	0.30 (3.40)			0.17 (0.01)			0.14 (1.85)			0.39 (0.01)		
**STEP 7**												
Intercept	1.90	0.42		23.47	2. 92		13.95	5.27		−5.32	3.09	
Age	−0.01	0.01	−0.30^*^	0.01	0.04	0.05	0.09	0.07	0.21	0.06	0.04	0.57
Gender	0.01	0.10	0.00	0.04	0.67	0.01	0.51	1.21	0.05	−2.62	0.80	−2.62^***^
Nonvb. intelligence	−0.01	0.01	−0.05	−0.25	0.10	−0.34^*^	−0.11	0.18	−0.09	0.26	0.12	1.07^*^
Anxiety	0.00	0.00	0.00	−0.01	0.03	−0.07	0.01	0.04	0.03	−0.06	0.03	−0.85
Vb. STM (T1)	0.09	0.14	0.15	−0.56	0.98	−0.15	−1.18	1.76	−0.18	2.90	1.19	2.17^*^
Vs. STM (T1)	−0.18	0.09	−0.27^+^	0.14	0.64	0.03	1.69	1.15	0.22	−0.24	0.68	−0.16
Vb. STM (T2)	−0.14	0.17	−0.21	2.20	1.14	0.52^+^	1.86	2.07	0.25	−2.22	1.28	−1.53
*R*^2^(Δ*F*)	0.30 (0.66)			0.22 (3.71^+^)			0.15 (0.81)			0.42 (3.38)		
**STEP 8**												
Intercept	1.96	0.42		23.19	2.93		13.05	5.17		−5.12	3.11	
Age	−0.01	0.01	−0.30^*^	0.01	0.04	0.04	0.09	0.07	0.19	0.05	0.04	0.58
Gender	−0.01	0.10	−0.01	0.07	0.67	0.01	0.59	1.18	0.06	−2.66	0.81	−2.66^***^
Nonvb. intelligence	0.00	0.01	−0.01	−0.27	0.10	−0.36^*^	−0.18	0.18	−0.14	0.27	0.13	1.11^*^
Anxiety	0.00	0.00	0.00	−0.01	0.02	−0.07	0.01	0.04	0.03	−0.06	0.03	−0.85
Vb. STM (T1)	0.15	0.14	0.26	−0.80	1.00	−0.22	−1.93	1.76	−0.30	2.97	1.21	2.23^*^
Vs. STM (T1)	−0.08	0.11	−0.11	−0.24	0.74	−0.05	0.41	1.30	0.05	−0.06	0.81	−0.04
Vb. STM (T2)	−0.18	0.16	−0.27	2.38	1.16	0.56^*^	2.44	2.04	0.33	−2.29	1.30	−1.58
Vs. STM (T2)	−0.17	0.09	−0.28	0.65	0.63	0.16	2.14	1.10	0.31	−0.28	0.66	−0.20
*R*^2^(Δ*F*)	0.35 (3.54)			0.23 (1.06)			0.21 (3.78)			0.43 (0.18)		

β, Standardized regression coefficient. For shifting, β was obtained by standardizing the continuous predictors, and Cox and Snell's R^2^ was calculated. T1, first time assessment; T2, second time assessment. Vb., verbal; Vs., visuospatial, RC, response conflict. The baseline gender is male.

+ *p < 0.06*,

* *p < 0.05*,

** *p < 0.01*,

*** p < 0.001.

#### Working memory

Individual differences in age, non-verbal intelligence, and anxiety differently accounted for children's WM variance. Accordingly, the first predictor considered, age, accounted for the largest proportion of variance explained in both verbal and visuospatial WM, while the addition of gender in the second step did not benefit the models. Non-verbal intelligence improved significantly only the model of verbal WM span. Further, individual differences in anxiety were associated to significant changes in the amount of variance explained in both WM spans, beyond the contributions of children's age and non-verbal intelligence scores. Overall, domain-specific STM measured at T1 was a very good predictor of the respective domain-specific WM, explaining as much as 18% of variance in verbal WM performance, after considering the effects of age, gender, non-verbal intelligence, and trait anxiety. On the other hand, domain non-specific STM did not improve either WM model. Controlling for previous (T1) STM spans, the addition of concurrent domain specific (T2) STM measures did not significantly improve the verbal WM model, but had a small significant effect on visuospatial WM. However, multicollinearity (*VIFs* as high as 5.6) between the STM spans at the two time points made it difficult to make inferences about individual (STM at T1 or at T2) predictors. We were primarily interested in the ability to predict WM based on STM at T1, therefore, we relied on the models in the sixth step of the hierarchical regressions to quantify this relationship (see Table 2).

**Figure 4:**
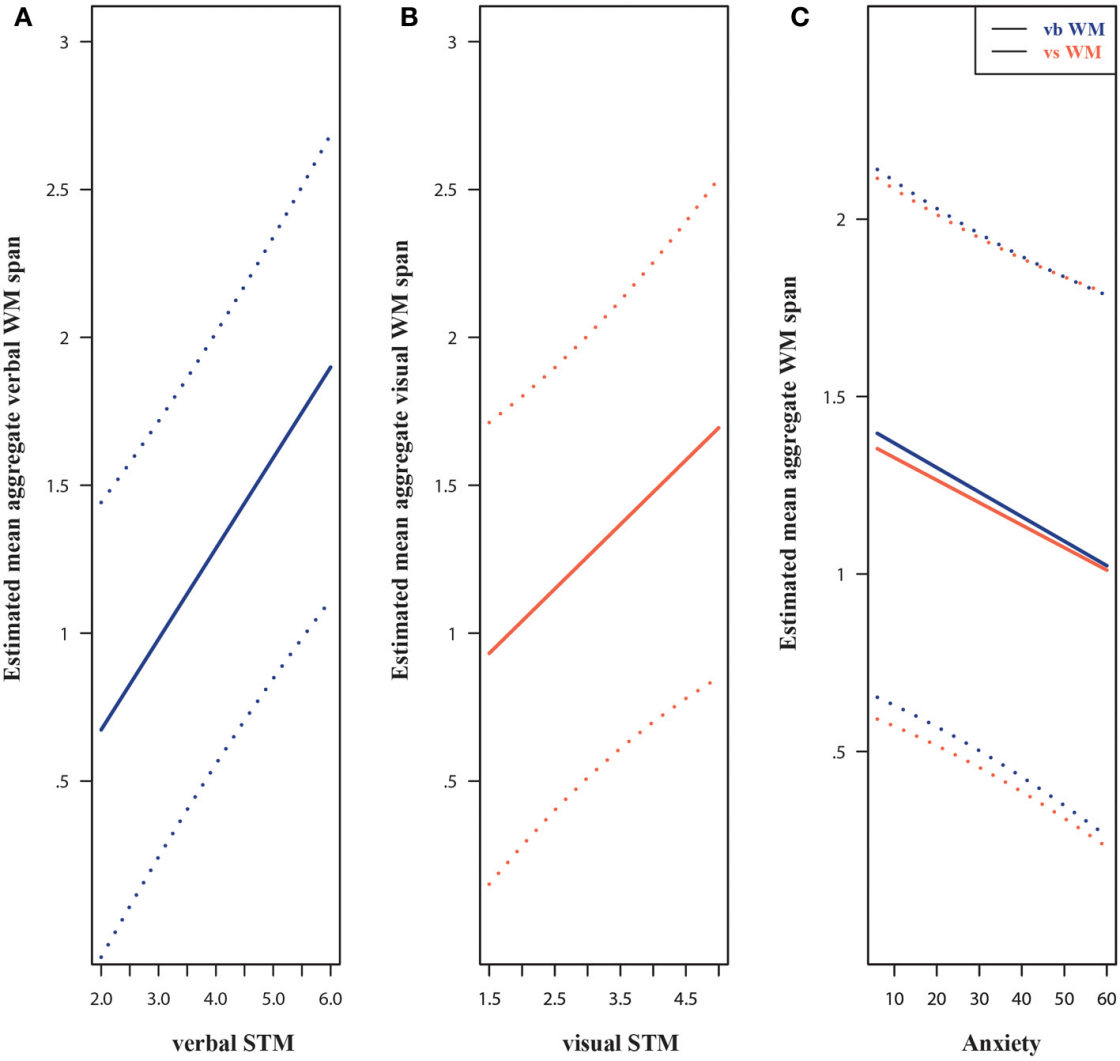
**Estimated regression lines and 95% Prediction Intervals for: verbal STM and verbal WM (A), visuospatial STM and visuospatial WM (B), and for trait anxiety and WM (C)**. Estimated means correspond to boys and all other (non-significant) continuous model predictors were set to the mean sample values. Vb., verbal; Vs., visuospatial; STM, short-term memory; WM, working memory.

In the final verbal WM model, the best predictor was verbal STM; a one point increase in verbal STM was associated with a change of 0.31 (95% CI from 0.17 to 0.44) in the estimated mean verbal WM span (see Figure 4A) keeping all other predictors constant. Also, higher non-verbal intelligence scores were linked to higher verbal WM performance, *b* = 0.04, *SE* = 0.01, *p* = 0.01. Children with higher anxiety scores tended to have lower verbal WM spans, *b* = −0.007, *SE* = 0.003, *p* = 0.05. This result is illustrated in Figure 4C. Lastly, age, gender, and visuospatial STM (T1) were not significant in the final model.

Similarly, in the final model for visuospatial WM performance (step 6), visuospatial STM was the only significant predictor; a one point increase in STM span was associated with a 0.22 increase (95% CI from 0.04 to 0.40) in estimated mean WM span (see Figure 4B), given the other model predictors. The age, gender, non-verbal intelligence, trait anxiety levels, and verbal STM (T1) did not prove significant. According to the bootstrapped *R*^2^, denoting the ratio of explained variance, the verbal WM model (bootstrapped *R*^2^ = 0.50, 95% BCa CI from 0.35 to 0.68) performed relatively well. However, the visuospatial WM model did not match this performance, bootstrapped *R*^2^ = 0.32 (95% BCa CI from 0.19 to 0.52).

#### Inhibition

***Response suppression***. The hierarchical regression revealed that beyond the first step (age), no other variable improved the model for response suppression (see Table 3). This could be explained by a lack of variability in the outcome measure, nearing a ceiling effect. The effect of age remained significant in the model including gender, non-verbal intelligence, anxiety and verbal STM (T1), but was insignificant in subsequent models. A description of the relationship between age and response suppression is presented in section Cross-sectional effects of age.

***Response conflict***. The first significant improvement in the model for *motor response conflict* (Knock and Tap) came with the addition of verbal STM at T1 in the fifth step, although the addition of non-verbal intelligence (step 3) was marginally significant. In the final model (step 6), only verbal STM remained significant when controlling for age, gender, non-verbal intelligence, anxiety, and visuospatial STM at T1, *b* = 1.07, *SE* = 0.49, *p* = 0.030. The scarcity of good predictors is probably related to the fact that, on this task, the performance of the majority of children was very good and there was little variability in the outcome measure. The overall model (step 6) performed poorly, bootstrapped *R*^2^ = 0.12 (95% BCa CI from 0.05 to 0.26).

For *verbal response conflict*, the addition of any variables after the first step (age) proved inconsequential in improving the model fit. Age continued to be a good predictor in the model containing age, gender, non-verbal intelligence, and verbal and visuospatial STM at T1 (step 6), *b* = −0.01, *SE* = 0.01, *p* = 0.030. Despite this association, the verbal response conflict model performed less well overall as compared to both WM models, bootstrapped *R*^2^ = 0.21 (95% BCa CI from 0.11 to 0.40).

#### Shifting

**Figure 5:**
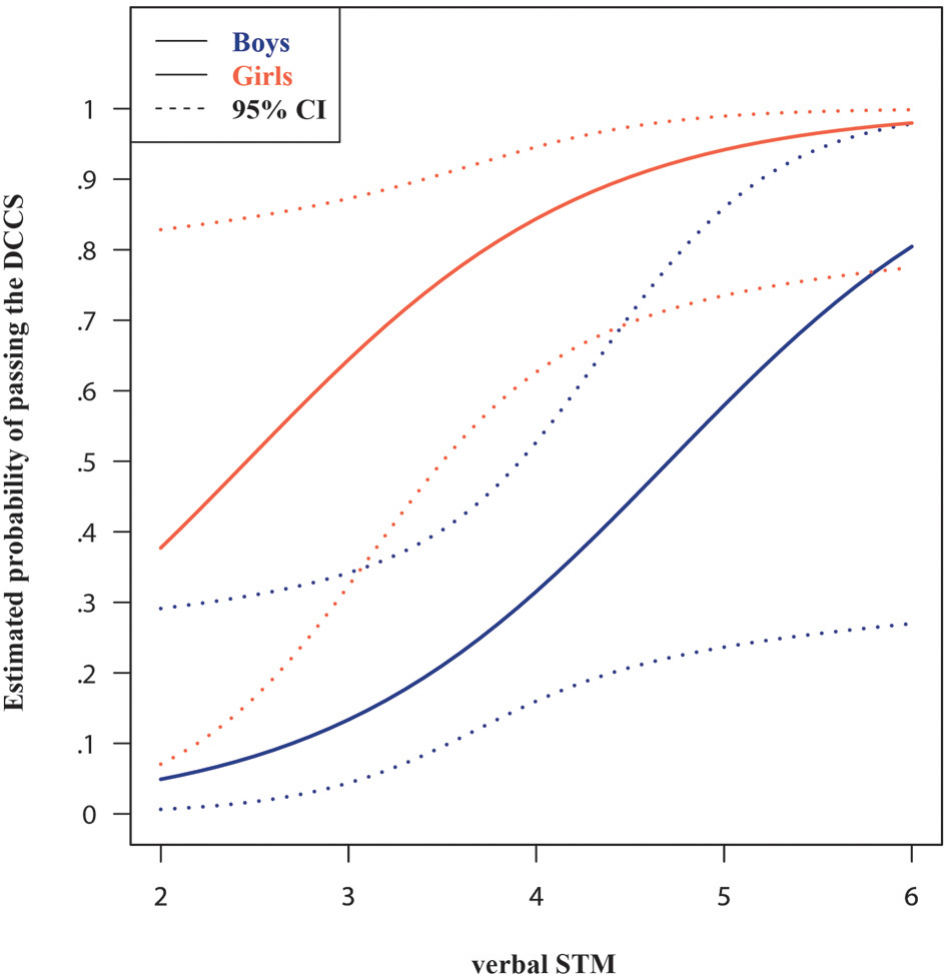
**Estimated probabilities of success on the shifting (DCCS) task, as a function of verbal STM span and gender**. Estimated means correspond to boys and all other (non-significant) continuous model predictors were set to the mean sample values. STM, short-term memory.

The probability of passing the DCCS test provided a measure of children's shifting performance. The hierarchical regression (see Table 3) revealed that besides age, the addition of gender, anxiety, and verbal STM at T1 improved previous models. In the final model (step 6), age was no longer significant, alongside non-verbal intelligence, trait anxiety and visuospatial STM at T1. However, keeping all else constant, verbal STM span at T1 was a useful predictor of shifting performance. The estimated probability of success was larger for children with better verbal STM performance as the odds of success were 2.99 times (95% CI from 1.06 to 8.40) larger for children who had verbal STM spans larger by one unit than their peers. The DCCS was the only measure for which we observed gender differences. The odds ratio of success for the girls relative to the boys was 11.74 (95% CI from 2.70 to 51.04), given the same age, non-verbal intelligence, anxiety, and STM spans. A graphical representation of the predicted probabilities of success as a function of gender and verbal STM at T1 is provided in Figure 5. The performance of the model in terms of (Cox and Snell's^1^) *R*^2^ was comparable to the one of the WM models, bootstrapped *R*^2^ = 0.36 (95% BCa CI from 0.24 to 0.53).

We were also interested in whether there were differences in the post-switch DCCS performance between sad and happy stimuli. A McNemar's test failed to show any differences related to the emotionality of the faces, χ^2^(1) = 0.55, *p* = 0.46. Further, we wanted to explore whether anxiety influenced DCCS trials with different expressions to a similar extent using a more sensitive measure of performance based on accuracy, rather than a pass/fail criterion. Two Poisson regressions were carried out for each expression including gender, anxiety, and verbal STM as predictors. Anxiety was a significant predictor for sad post-switch trials, *b* = −0.014, *SE* = 0.007, *p* = 0.05, but not for happy post-switch trials, *b* = −0.005, *SE* = 0.006, *p* = 0.45.

## Discussion

Our study addressed two major research questions. The first one was developmental, concerning the interrelationships between early levels of STM performance and subsequent levels of the same ability, assessing their predictive value for three EF dimensions: WM, inhibition, and shifting. The second one was an individual differences question, and it concerned the predictive value of early levels of anxiety on subsequent EF, controlling for the influence of other individual differences in age, gender, or non-verbal intelligence. In the literature, both anxiety and STM, have been linked to attention control (dys)functions, making it plausible to assume that attention control could represent a mechanism responsible for their association with EF performance.

### Early EF development and its precursors

A preliminary analysis of inter–task *correlations* revealed stronger relationships between measures designed to tap the same underlying memory component, confirming domain specificity. The verbal and visuospatial scales correlated better for WM than for STM, confirming that WM measures using different stimuli actually capture more common underlying cognitive processes than STM tasks. This fits nicely with the suggestion that controlled attention works to keep task-relevant information active in WM across a variety of stimulus modalities (Engle et al., 1999). Interestingly, reanalyzing correlations among the three inhibitory control measures, we noted that the proposed dissociation between response suppression and response conflict measures (e.g., Friedman and Miyake, 2004; Espy and Bull, 2005) was not fully validated. More specifically, while the associations between the verbal and motor response conflict were poor, scores on response suppression were significantly related to verbal response conflict. The lack of a significant association between the two response conflict measures could have different explanations, including the different outcome measures (accuracy vs. inverse efficiency), the use of different stimuli (verbal vs. visuospatial), or a truly modest coherence between various inhibitory control measures in young children (see Lerner, 2012; Van der Ven et al., 2012; Cheie et al., 2014). However, although expected for this age range (e.g., Willoughby et al., 2012b), the high levels of performance reached in most inhibitory task preclude us from drawing strong conclusions regarding the independence or interdependence of various inhibitory control measures.

The cross-sectional analysis of the evolution of STM and EF abilities within this developmental period revealed different growth patterns for the various outcomes measured. The mean *verbal STM span* improved over the course of 3 years by roughly one unit, meaning that while the youngest children (aged 3) had a mean maximal span of 3, the oldest ones (aged 6) had a mean maximal span of 4. Verbal STM performance during this period most likely reached its peak sometime between the ages of 4 and 5 years, and then in the transition to 6 years progress stagnated. This confirms previous research indicating that performance in verbal STM tasks levels off sooner than in visuospatial ones, although the exact level at which this plateau occurs is placed later, at about 10–11 years (Alloway et al., 2006). This suggests that our findings might indicate simply a transitory slowing down of verbal STM progress in the late preschool years. However, this does not imply that there is no within-individual gain in verbal STM, as such gains were evident in our study, over the 9 month period, and did not vary as a function of age. Moreover, it is plausible that over this apparent stagnation period, lower performing children may still continue to improve so as to match their peers. This statement can be supported by the negative correlation between STM at the first measurement point and the within-participant gain in STM.

The *development of visuospatial STM* was more gradual, performance increasing linearly within the age range recorded in the study (between 3 and 6 years of age). This parallels previous proofs of a steady increase in performance on tests that employ visual material that is not phonologically recordable (e.g., Pickering et al., 2001). A storage hypothesis has been proposed (Logie and Pearson, 1997), suggesting the increase in the capacity of the visuospatial sketchpad. Alternatively, an increasing involvement of the central executive has been suggested via more effective strategies or long-term memory knowledge deployment (Pickering et al., 2001). However, a better rate of attention shifting between locations could also be responsible for the increase in spatial span (Smith and Jonides, 1997). Using a similar measure across tasks (the aggregate span) allows us to directly contrast absolute levels of performance on the verbal compared to the visuospatial STM. Evident from the descriptive analyses, visuospatial STM in the oldest children (6–7 years) did not match comparable levels of verbal STM at a much younger age (4–5 years). This confirms the well-documented inferior visuospatial span compared to the verbal one in preschool children (e.g., Pickering et al., 1998; Alloway et al., 2006). The fact that these tasks are experienced as more difficult is consequential for their greater involvement in some EF tasks discussed below, confirming that visuospatial STM tasks draw more executive resources than the verbal STM measures (Miyake et al., 2001; Alloway et al., 2006).

Age-related improvements regarding children's *WM performance* appeared to be more gradual, similar to previously identified trends (Alloway et al., 2006). The mean aggregate span increased with roughly half a unit over the course of three years on both verbal and visuospatial WM measures. During the developmental course of WM, it appears that domain-general processing mechanisms interact with domain-specific storage components leading to a gradual progress (Bayliss et al., 2003, 2005; Alloway et al., 2006) also documented in the current study. Modest age-related improvements in performance also occurred on the *motor response conflict* and the *response suppression* task. However, there were no age-related improvements on the accuracy measure from the verbal response conflict task, but this could be explained by the fact that children's performance reached ceiling levels.

The probability of passing the DCCS also increased with age, yet even for children in the older age group (5–6 years) performance did not reach maximal accuracy (only 58% of 5-year olds achieved perfect post-switch performance. Interestingly, we found poorer levels of performance employing an emotional shifting task compared to previous results with the standard version of the DCCS in this age range. We believe that the explanation might relate to either (1) the greater impact of emotional expression as a categorizing criterion and in the resulting *negative priming effect,* or (2) the greater *perceptual conflict* between the two dimensions (color and emotional expression) induced by our stimuli. Related to the first explanation, Müller and Zelazo (2001) have proposed that a negative priming effect might be generated in the DCCS task by the need to inhibit a dimension (here, the emotional expression) in order to focus solely on the target dimension (i.e., color), and then to “undo” this initial inhibition during the second phase (i.e., when emotional expression becomes the target dimension). To be more specific, it is not that children have trouble with inhibiting this dominant dimension (in the pre-shift phase), but rather that they have difficulty disengaging this negative priming effect from the pre-shift set during the post-shift phase (Garon et al., 2008). This negative priming explanation could be tested in a future study by reversing the order of the dimensions (asking the child to categorize the items first by emotion, and then by color) which should theoretically reduce this effect. A second possible explanation relates to the higher degree of *perceptual conflict* elicited by the two dimensions during the pre-shift phase. The main distinction from the previous account is that it does not imply that sorting according to emotional expression was more salient, but that the target cards, were perceptually similar to a greater degree than, for instance, the boats and the rabbits. Apart from the color dimension which was clearly different, the emotional expression was related to a simple perceptual difference in the orientation of the mouth line. Future studies taking this explanation into account, could require the children to sort the cards according to the same two dimensions in the absence of the target cards, which has already been shown to improve performance (Perner and Lang, 2002), as no perceptual mismatch would be present. An alternative would be to separate the dimensions by placing them side by side on the card (as in Kloo and Perner, 2005).

### Concurrent and predictive effects

The results suggest that given only a time difference of 9 months between measurements, the overlap in STM spans was sufficiently large, such that adding concurrent STM performance to a model already containing the previous STM did not improve EF prediction to a significant extent. On one hand, this indicates the stability of the predictive relationships between STM and WM measures. It is possible, however, that given a larger time difference between measurements, a direct effect could have been observed. However, for visuospatial WM, the addition of the second time point STM was significant, suggesting that the impact of this variable during this 9 months interval is not fully accounted by its previous development.

In the final models, STM at the first assessment was the most consistent predictor of performance across the EF measures. The best model in terms of predictive ability was the verbal WM, where the variables in the model accounted for over 50% of variance in the outcome, about a third of explanatory power being attributed to verbal STM. The models for visuospatial WM and shifting had a somewhat poorer predictive performance (only about 30–35% of variance was explained) and models for inhibition were inadequate for prediction purposes (20% or less of variance was explained). These are also the only models in which STM was a weak predictor, especially for response conflict, which diverged from previous findings by Espy and Bull (2005). However, it is important to note that in that study, children were divided into dichotomous High and Low digit span groups, while here a more refined continuous measure was used for both verbal and visuospatial STM performance. In our case, high verbal STM spans were indicative of good motor response conflict and shifting performance, while there were no links between STM spans and response suppression or verbal response conflict. While the associations between verbal STM span and motor response conflict parallel those obtained by Espy and Bull (2005), it is difficult to relate the results concerning the (absence of) associations between visuospatial STM, response suppression, and verbal response conflict to previous literature since the current experimental design is not directly comparable to any previous study with preschoolers. Hence, our results need to be validated in other samples before an explanation could be advanced. Also, the identified relationship between verbal STM and shifting performance warrants further exploration, suggesting that cognitive flexibility - as reflected by the Em-DCCS—might be strongly dependent on children's ability to verbally encode and maintain relevant stimulus-related information for brief successive periods of time. Preliminary evidence supporting this idea comes from the same study of Espy and Bull (2005), in which preschoolers with higher memory spans outperformed those with lower memory spans in the flexibility condition of the Shape School task. In a more systematic investigation of the contribution of WM (actually measured with a verbal span task) to the costs of cognitive flexibility in preschoolers, Chevalier and collaborators (2012) showed that after 4½ years of age, verbal STM was associated with specific costs on the same Shape School task. This evidence was related to the crucial role of verbal memory in the identification and maintenance of task goals necessary for performance on the flexibility tasks (Blaye and Chevalier, 2011).

### The role of individual differences

Regarding *gender differences*, the only outcome on which such effects were found was shifting, as girls were significantly more likely to pass the DCCS task than boys. These results apparently are at odds with studies in which no gender differences were found in preschool children on the standard DCCS (e.g., Coldren and Colombo, 2009; Moriguchi et al., 2012). However, it is notable that some studies such as the one conducted by Wiebe and collaborators (2008) did find evidence of higher absolute levels of EF performance in preschool girls. Our results are also in line with studies reporting that preschool girls presented higher levels of effortful control (e.g., Olson et al., 2005; Raaijmakers et al., 2008). Also, the fact that our task involved operating with emotional material (categorizing a stimulus based on emotion) might have favored the performance of preschool girls, as this has been previously indicated by their better performance at decoding emotion from facial expressions (Boyatzis et al., 1993) and their faster emotional judgments after neutral ones in a shifting context (Mocan et al., 2014).

*Non-verbal intelligence* scores were linked to superior WM spans, but did not have an impact on other EF performances. These results correspond to many adult and developmental studies showing that WM performance is closely related to intelligence scores (e.g., Fry and Hale, 2000; Colom et al., 2003), as both types of tasks employ attentional control (Engle, 2010). Moreover, non-verbal intelligence was modestly correlated with STM spans, but not with larger gains in children's STM spans. These results are in line with developmental findings suggesting that when the common variance between WM and STM is controlled, the residual WM factor is linked to children's intelligence, whereas the residual STM factor is not (Engel de Abreu et al., 2010). Hence, the high inter-subject variation observed for WM could be a reflection of the fact that WM performance relies on individual differences beyond STM performance. Taken together, our findings suggest that the link between intelligence and WM performance in young children could be mainly explained by the cognitive control mechanisms employed in WM tasks, and not by the storage component of such measures.

Regarding the impact of *trait anxiety* upon children's EFs, our hypotheses were partially confirmed. It is important to note that verbal STM performance at either time point correlated with initial levels of anxiety, and there was no significant link between anxiety and visuospatial STM. These results resonate well with the lack of anxiety-related effects on visuospatial STM found in a previous study with preschoolers (Visu-Petra et al., 2011). ACT (Eysenck et al., 2007) predicts that such effects should be less visible on the accuracy scores of tasks employing lower levels of attentional control, and more evident in efficiency measures, which were not available for our STM measurements. In line with this prediction and our current results, Visu-Petra and collaborators (Study 1, 2011) found no impact of trait anxiety upon preschoolers' STM accuracy, yet on the verbal storage tasks, there was a detrimental effect upon children's processing efficiency (i.e., duration of preparatory intervals). Moreover, it is important to note that higher anxiety at the start of the experiment did not result in lesser gains in STM performance, measured as the difference between the two STM measures.

With regards to children's performance on a task with similar levels of executive demands, we found that response suppression scores were not significantly affected by anxiety. However, contrary to our expectations, anxiety did not have a negative effect on either of the two response conflict measures, which presented higher levels of executive demands. There is very limited empirical evidence for such a relationship during early childhood for typically developing children (e.g., Cheie et al., 2014), as the studies have been mostly conducted with pediatric anxiety (e.g., Mueller et al., 2012). However, our negative findings should be regarded with caution considering the high levels of performance registered on all inhibitory control measures, as well as the fact that two of these measures provided only accuracy, and no efficiency outcomes.

As anticipated, trait anxiety was a significant predictor for WM components. The association between trait anxiety and verbal WM was significant even when controlling for other individual differences and STM spans. While the negative effect of anxiety was non-significant in the final model for visuospatial WM, the fact that anxiety significantly predicted performance in a previous model in which verbal (i.e., domain non-specific) STM was omitted is suggestive of the importance of this predictor for visuospatial WM. However, based on the magnitude of the effects, it is most likely that anxiety only affects WM to a practically relevant extent if the children are situated toward the upper end of the non-clinical spectrum. These results are in line with the ACT's predictions (Eysenck et al., 2007) regarding anxiety's deleterious impact upon updating, and correspond to the developmental empirical evidence highlighting such effects (e.g., Hadwin et al., 2005; Owens et al., 2008; Ng and Lee, 2010; Visu-Petra et al., 2011). To our knowledge, it is the first time that a detrimental impact of anxiety on a visuospatial WM measure is observed in young children. These findings are at odds with Visu-Petra and collaborators' (2011) results, which revealed no significant impact of anxiety upon young children's visuospatial updating performance. Yet, in adults, several studies have found individual differences in (threat-induced) state anxiety to account for performance variations in visuospatial WM (e.g., Shackman et al., 2006). Given the limited literature regarding the anxiety-visuospatial WM relationship in young children, replications of this effect are needed to shed light in this specific domain, especially considering the abovementioned idea of the higher executive load (and increased difficulty) experienced by children when performing the visuospatial memory tasks.

Anxiety also impacted preschoolers' shifting performance when controlling for individual differences in age, gender, and non-verbal intelligence. However, the effect became non-significant with the addition of STM measures. This result apparently fails to confirm our hypothesis and that of the ACT in predicting anxiety's detrimental effects in tasks employing set-shifting (Eysenck et al., 2007). On the other hand, taking a closer look at stimulus valence, the impact of anxiety on performance in the post-switching phase was restricted to children's perseverative errors in categorizing the sad (but not happy) faces according to the previous dimension (color). There is a documented general happy face advantage in recognizing even schematic facial expressions (Kirita and Endo, 1995), already visible in infants (Barrera and Maurer, 1981), which might have aided children's performance on this type of stimuli. However, we failed to replicate the facilitative effect of positive faces found in the study of Qu and Zelazo (2007). One crucial difference is that in the study by Qu and Zelazo (2007), in the happy/sad/neutral faces conditions, children were not required to perform any judgment based on emotion, but solely on age and gender. Therefore, the emotion of the face was not the target of the evaluation, as it was in the current study. What could have impaired high-anxious children's performance in assessing the sad faces according to emotion, and made them continue in sorting them according to color? One important clue could come from the systematic analysis, performed by Kirita and Endo (1995) of how emotion displayed by schematic faces is recognized. Their study indicated that while happy (schematic) faces appeared to be recognized holistically, sad faces were more likely to be recognized in an analytical mode. In this respect, their results showed that sad faces were less disrupted by being presented in an inverted mode, as compared to the happy faces, for which the advantage completely disappeared in this inverted mode. It is plausible that this analytical mode of processing in recognizing emotion might have imposed greater executive demands, which have selectively disrupted high-anxious children's shifting performance. An alternative explanation would relate to their specific processing of negative emotional information conveyed by a sad face which would lead to a phenomenon of “cognitive avoidance” (Cloitre and Liebowitz, 1991) and probably to a re-focusing on perceptual aspects of performance such as stimulus color. However, a replication of this effect in an independent sample is required before attempting to distinguish between such potential explanations. Taken together, our findings reveal the crucial importance of taking individual differences (gender, intelligence, trait anxiety) into account when studying EF in young children, considering that such differences might influence, and might themselves be influenced, by individual progress in executive performance.

### Limitations

There are several limitations which call into question the generalizability of our findings. Some of the limitations are methodological, induced by the study design, sample and procedure, while others are more related to the analytical approach—itself limited by the methodological constraints. More specifically, one of the main methodological limitations induced by looking at a developmental period characterized by intensive changes in all the assessed dimensions is that performance for the older children will inevitably reach *ceiling* levels of performance. This effect was found in our study to affect mostly measures of inhibition and shifting, similar to previous findings over the same age range (e.g., Lerner, 2012; Willoughby et al., 2012b). Another important methodological limitation was induced by the lack of a processing speed measure, this variable being causally related to changes in both memory span and executive functioning (Kail and Salthouse, 1994; Salthouse et al., 1998; Chuah and Mayberry, 1999).

A significant limitation makes us cautious with regards to directly incorporating current results in the ACT framework (Eysenck et al., 2007). As the theory predicts that anxiety-related worrisome thoughts interfere with the current task performance of an individual, the absence of a direct state anxiety measure (at both T1 and T2) precludes us from having clear-cut conclusions in this respect. However, using just a trait anxiety measure can be explained by the fact that in preschoolers, self-report measures of state anxiety are difficult to obtain in a reliable manner (Schniering et al., 2000). At the same time, studies also report that individuals with high levels of trait anxiety also experience higher levels of state anxiety in potentially stressful situations, such as performance evaluation for cognitive tasks (Lau et al., 2006). Nevertheless, trait anxiety was only evaluated at T1 and, while it could have remained stable within the T1-T2 interval, this was not directly verified in our study, this jeopardizing the incorporation of our findings in the ACT framework.

Considering the limitations of our analytic approach, it is important to note when discussing correlations among EF measures that these can arise from true similarities in the mechanisms underlying performance, but can also be confounded by common age-related effects and by shared method variance which can lead to spurious overlaps (e.g., reliance on verbal skills or on processing speed). These can only be eliminated by using multiple tasks tapping the same construct and relying on latent variable analysis to exclude such measurement error (Willoughby et al., 2012b). We only accomplished this objective to a certain extent, especially in terms of STM and WM measures, but to a lesser extent in terms of inhibition and especially of shifting performance. Also, other impediments eliminated the possibility of a latent variable analysis were the array of distributions of variables (ceiling effects noted above), and the limited sample size available at both time points.

## Conclusions and future directions

The aforementioned limitations notwithstanding, there are some particular strengths of the current study. These are reflected by the use of repeated assessments conducted at a young age and related to subsequent levels of performance, the choice (wherever possible) of multiple tasks to assess each construct and its subcomponents, and the often overlooked analyses of the impact of individual differences (in age, non-verbal intelligence, and anxiety).

First, regarding the developmental prerequisites of EF, STM appears to be a reliable and stable predictor during this interval (especially of WM and especially for the same stimulus modality). A cautionary note relates to several studies with preschoolers which have investigated WM by using tasks purported to measure STM (Hughes and Ensor, 2007; Wiebe et al., 2008; Noël, 2009). Very early during development such an overlap might be justified by the high demands posed by a memory span task for very young children (Reznick, 2007). However in older preschoolers our study concurs with other investigations (e.g., Alloway et al., 2006; Lerner, 2012) in revealing the necessity to delineate between STM tasks and WM tasks and to focus on the latter as a more adequate measure of EF. Repeated assessments of STM are necessary in order to identify the potential dynamics of this interrelationship and their presumed common reliance on attention control/processing speed improvements. We did not replicate the postulated distinction between response suppression and attention control mechanisms. It could be that at a young age they are truly undifferentiated, or that our tasks failed to impose a similar level of difficulty required in order to analyze inter-task correlations (see also Carlson, 2005).

Regarding the impact of individual differences, we found specific links between gender and shifting, between non-verbal intelligence and WM, and a potential link between trait anxiety and verbal/visuospatial WM. While some of these results fit nicely and extend the theoretical frameworks proposed in the literature with adults (e.g., the ACT; Eysenck et al., 2007), they need substantial replication in larger independent samples and repeated assessments of individual differences over time. For instance, it would be relevant to measure anxiety at more than one time point in order to observe if past levels of anxiety affect performance beyond current levels, suggesting an early impact of anxiety on information-processing patterns, probably as a consequence of the enhanced plasticity of young children's threat-processing circuitry (Pine, 2007). Again, more intermediary time points are also needed in order to fully grasp the reciprocal interactions between cognitive, emotional, and (pre)dispositional factors during early development.

### Conflict of interest statement

The authors declare that the research was conducted in the absence of any commercial or financial relationships that could be construed as a potential conflict of interest.

## Footnotes

^1^Note that the maximum of the Cox and Snell pseudo **R*^2^ is lower than 1 (1− L(M_Intercept_)^2/N^)*.
