# Supplementary material for: Longitudinal and concurrent links between memory span, anxiety symptoms, and subsequent executive functioning in young children
Source: Front Psychol. 2014 May 16;5:443. doi: 10.3389/fpsyg.2014.00443 (PMC4032945; doi:10.3389/fpsyg.2014.00443)
Supplement: Supplementary file 1 [file DataSheet1.DOC]

***Appendix.***

Correlation matrix of the raw measures.

|  | Age | Anxiety | Nonvb.  intel. | Word  span T1 | Digit  span T1 | Corsi  span T1 | Word  span T2 | Digit  span T2 | Corsi  span T2 | Count  span | Back  span | Listen  span | Odd  one-out | Mr. X | Statue | Stroop |
| --- | --- | --- | --- | --- | --- | --- | --- | --- | --- | --- | --- | --- | --- | --- | --- | --- |
| Anxiety | -.21 |  |  |  |  |  |  |  |  |  |  |  |  |  |  |  |
| Nonvb. intel. | .36** | -.09 |  |  |  |  |  |  |  |  |  |  |  |  |  |  |
| Word span T1 | .30* | -.27* | .04 |  |  |  |  |  |  |  |  |  |  |  |  |  |
| Digit span T1 | .27* | -.27* | .05 | .83** |  |  |  |  |  |  |  |  |  |  |  |  |
| Corsi span T1 | .49** | -.02 | .33** | .25* | .33** |  |  |  |  |  |  |  |  |  |  |  |
| Word span T2 | .43** | -.23 | .26* | .71** | .80** | .38** |  |  |  |  |  |  |  |  |  |  |
| Digit span T2 | .29* | -.25* | .34** | .75** | .84** | .24* | .76** |  |  |  |  |  |  |  |  |  |
| Corsi span T2 | .40** | -.10 | .26* | .31* | .33** | .62** | .22 | .26* |  |  |  |  |  |  |  |  |
| Count span | .45** | -.27* | .41** | .47** | .53** | .40** | .63** | .52** | .36** |  |  |  |  |  |  |  |
| Back span | .48** | -.26* | .50** | .51** | .55** | .32* | .62** | .55** | .32* | .65** |  |  |  |  |  |  |
| Listen span | .25 | -.43** | .19 | .43** | .41** | .24 | .39** | .31* | .37** | .57** | .53** |  |  |  |  |  |
| Odd-one-out | .43** | -.36** | .28* | .35** | .41** | .49** | .38** | .43** | .48** | .47** | .39** | .29* |  |  |  |  |
| Mr. X | .32* | -.17 | .44** | .28* | .25 | .38** | .40** | .37** | .46** | .40** | .42** | .34** | .55** |  |  |  |
| Statue | .32** | -.06 | .07 | .20 | .11 | .31** | .18 | .17 | .38** | .18 | .20 | .07 | .17 | .07 |  |  |
| Stroop | -.48** | .09 | -.28* | -.19 | -.21 | -.47** | -.27* | -.26* | -.46** | -.39** | -.45** | -.23 | -.27* | -.23 | -.45** |  |
| Knock & Tap | .10 | -.12 | -.12 | .29* | .34** | .11 | .38** | .34** | .14 | .36** | .28* | .41** | .10 | .06 | .32** | -.22 |

Note. Nonvb. intel. = nonverbal intelligence; Count = counting; Back = backwards; Listen = listening; **p* <.05; ***p* <.01
